# Pursuing dementia detection for better prevalence estimation with artificial intelligence and traditional modelling techniques in electronic health data

**DOI:** 10.1002/alz.090057

**Published:** 2025-01-09

**Authors:** Taya Collyer, Ming Liu, Richard Beare, Nadine Andrew, Jenni Ilomaki, Simon Bell, Walter A. Rocca, Jennifer St. Sauver, Amanda G Thrift, David Ung, Alicia Lu, Kristy Siostrom, Chris Moran, Helene Roberts, Elizabeth Le, John Clark Kennedy, Velandai K Srikanth

**Affiliations:** ^1^ Peninsula Clinical School, Central Clinical School, Monash University, Melbourne, VIC Australia; ^2^ Monash University, Melbourne, VIC Australia; ^3^ Murdoch Children’s Research Institute, Royal Children’s Hospital, Melbourne, VIC Australia; ^4^ Mayo Clinic, Rochester, MN USA; ^5^ Monash University, Melbourne Australia

## Abstract

**Background:**

Population dementia prevalence is traditionally estimated using cohort studies, surveys, routinely‐collected administrative data, and registries. Hospital Electronic Health Records (EHRs) are comprised of rich structured and unstructured (text) clinical data that are underutilised for this purpose.

We aimed to develop a suite of algorithms using routinely‐collected EHR data to reliably identify cases of dementia, as a key step towards incorporating such data in prevalence estimation. Towards this, we developed a novel predictive framework integrating data‐science and biostatistical methods.

**Method:**

Training data were sourced via the National Centre for Healthy Ageing (NCHA) Data Platform, a linked, curated, EHR‐derived data warehouse. Individuals within the platform catchment aged >60 years with confirmed dementia were identified through hospital specialist dementia clinics. A comparison group of individuals aged >60years with EHR records without dementia was recruited from the community.

A panel of clinical experts (Neurology, Geriatric Medicine) informed variable and concept selection and guided data cleaning efforts within both streams. Algorithms were developed via two work‐streams; a traditional biostatistical approach to fit logistic regression models using structured data elements, and a data science stream used Natural Language Processing (NLP) to fit models to the unstructured (text) parts of the EHR, for the same individuals.

**Result:**

Of 568 individuals (362 with dementia), 434 had clinical notes available. In the data science stream using unstructured data, among a range of NLP derived models, the Random Forest classifier performed best in assigning dementia status, with Area Under the Curve (AUC) 0.95, specificity 90.2% and sensitivity 88.4%. In the biostatistics stream, 15 structured variables were included in the final model, covering demographics, health service attendance, medications, and ICD‐10 Codes, with AUC 0.94, specificity 85.9% and sensitivity 85.6%.

**Conclusion:**

Artificial intelligence techniques applied to unstructured electronic health data and guided by human clinical expertise may be powerful tools in capturing the presence of dementia, at least comparable to traditional techniques using structured data, and conferring practical and scientific advantages for dementia prevalence estimation. Future validation is required in less crisply delineated real‐world settings.

